# Spindle and kinetochore-associated complex subunit 3 could serve as a prognostic biomarker for prostate cancer

**DOI:** 10.1186/s40164-022-00337-3

**Published:** 2022-10-20

**Authors:** Dechao Feng, Weizhen Zhu, Xu Shi, Qiao Xiong, Dengxiong Li, Wuran Wei, Ping Han, Qiang Wei, Lu Yang

**Affiliations:** Department of Urology, Institute of Urology, West China Hospital, Sichuan University, Guoxue Xiang #37, Chengdu, 610041 Sichuan People’s Republic of China

**Keywords:** Spindle and kinetochore associated complex subunit 3, Prostate cancer, Biomarker, Enrichment analysis, Targeted therapy

## Abstract

Spindle and kinetochore-associated complex subunit 3 (SKA3) is a microtubule-binding subcomplex of the outer kinetochore that is required for proper chromosomal segregation and cell division. However, little is known regarding the probable mechanism of SKA3, particularly in terms of prostate cancer (PCA) progression. Multiple databases, including TCGA and GTEx, were utilized to examine the expression of SKA3 in PCA patients and to shed light on the clinical significance and potential mechanism of SKA3 in the onset and progression of PCA. The biological function of SKA3 was evaluated in vitro using RT–qPCR and the CCK8 assay. For statistical analysis, the R 3.6.3 software and its associated packages were utilized. SKA3 was shown to be considerably elevated in PCA patients and was linked to a shorter progress free interval (PFI). Furthermore, we discovered that SKA3 mRNA expression was higher in PCA cells than in normal cells, and inhibition of SKA3 could clearly reduce PCA cell proliferation using the CCK8 assay. Finally, SKA3 could be used as a predictive biomarker in PCA patients.

**Supplementary Information:**

The online version contains supplementary material available at 10.1186/s40164-022-00337-3.

To the editor,

The age-standardized incidence rate and death rate of prostate cancer (PCA) in 2019 were 17.39 and 15.28, respectively [1]. The majority of PCA burden was found in older men [2]. By 2044, the aging population will account for approximately 20.8% of the total population [3]. PCA is an age-related disease whose social conundrum can be compounded by global population aging [4]. Clinical heterogeneity in PCA can be illustrated by regional and clonal genetic variety [5]. With the completion of the cancer genome atlas (TCGA), researchers will have access to a mix of genetic and clinical characteristics to approach precision medicine at several levels. Spindle and kinetochore associated complex subunit 3 (SKA3) is a microtubule-binding subcomplex of the outer kinetochore that is essential for proper chromosome segregation and cell division [6–8]. SKA3 knockdown activates the spindle assembly checkpoint, causing sister chromatid cohesion loss and mitotic arrest during metaphase [9]. Differential expression of SKA3 has been linked to the progression and prognosis of various malignant cancers, including laryngeal squamous cell carcinoma [9] and lung adenocarcinoma [10]. Furthermore, our earlier research found that SKA3 may contribute to poor overall survival, disease-specific survival, and progression-free survival in patients with renal papillary cell carcinoma [11]. There is, however, little published evidence about the putative molecular mechanism of SKA3 carcinogenesis in PCA. Based on several public datasets, we investigated the differential expression of SKA3, as well as its connection with clinicopathological characteristics and prognosis.

Multiple databases, including TCGA and GTEx, were utilized to examine the expression of SKA3 in PCA patients and to shed light on the clinical significance and potential mechanism of SKA3 in the onset and progression of PCA. The biological function of SKA3 was evaluated in vitro using RT–qPCR and the CCK8 assay. For statistical analysis, the R 3.6.3 program and its associated packages were utilized. The extra material contained the full-text article as well as comprehensive methodologies.

This study included 499 PCA samples and 52 tumor-adjacent tissues from the TCGA database. SKA3 expression was linked to advanced T stage, N1 stage, positive residual tumor, and a higher Gleason score (Table 1). SKA3 mRNA expression was higher in most tumors relative to normal tissues in the pan-cancer analysis of non-paired and paired samples, including PCA (Fig. 1A, B), which was similar with the results of the PCA dataset in the TCGA database (Fig. 1C, D). Furthermore, RT–qPCR revealed that SKA3 was more abundant in PCA cell lines than in normal prostate cell lines (Fig. 1E). The area under the curve (AUC) was 0.887 (95% CI 0.853–0.921), demonstrating that SKA3 may identify tumor from normal patients (Fig. 1F). The AUC in the subgroup analysis of T2–3 versus T4 was 0.750 (95% CI 0.589–0.910). (Fig. 1G). PCA patients with high SKA3 expression had a longer PFI than those with low expression (P0.001, Fig. 1H). Furthermore, patients with higher SKA3 had considerably shorter PFI when compared to their counterparts in the T3 stage, N0 stage, M0 stage, PSA4 ng/ml, positive residual tumor, and white (this signified white population) subgroup analysis (Fig. 1I–N). Using the RT–qPCR test, we discovered that SKA3 expression was downregulated after transfection of the three siRNAs on the proliferation ability of PCA cell lines (Fig. 1O). Furthermore, these siRNAs were able to dramatically inhibit the ability of PCA cells to multiply (Fig. 1P–T), particularly in cells with high levels of malignancy, such as C4-2B, PC3, and DU145, which was consistent with the clinical correlations of SKA3.

To date, no association between SKA3 and PCA patients has been observed. SKA3 mRNA expression was found to be significantly higher in most malignancies than in normal tissues in our investigation. Furthermore, we discovered that SKA3 mRNA expression was higher in PCA cells than in normal cells, and inhibiting SKA3 may obviously reduce PCA cell growth. Furthermore, those with higher SKA3 mRNA expression had a higher risk of advancement than those with lower SKA3 mRNA expression. Moreover, patients with T3 stage, N0 stage, M0 stage, PSA4 ng/ml, positive residual tumor, or white individuals were more likely to progress if they had high levels of SKA3 mRNA. These findings suggest that increased SKA3 expression may enhance the onset and progression of PCA and has a strong clinical connection. Based on the subgroup analysis, we also postulated a dose impact of SKA3 carcinogenesis.

In conclusion, SKA3 might involve in the progression of PCA and serve as a prognostic biomarker for PCA patients.

**Fig. 1:**
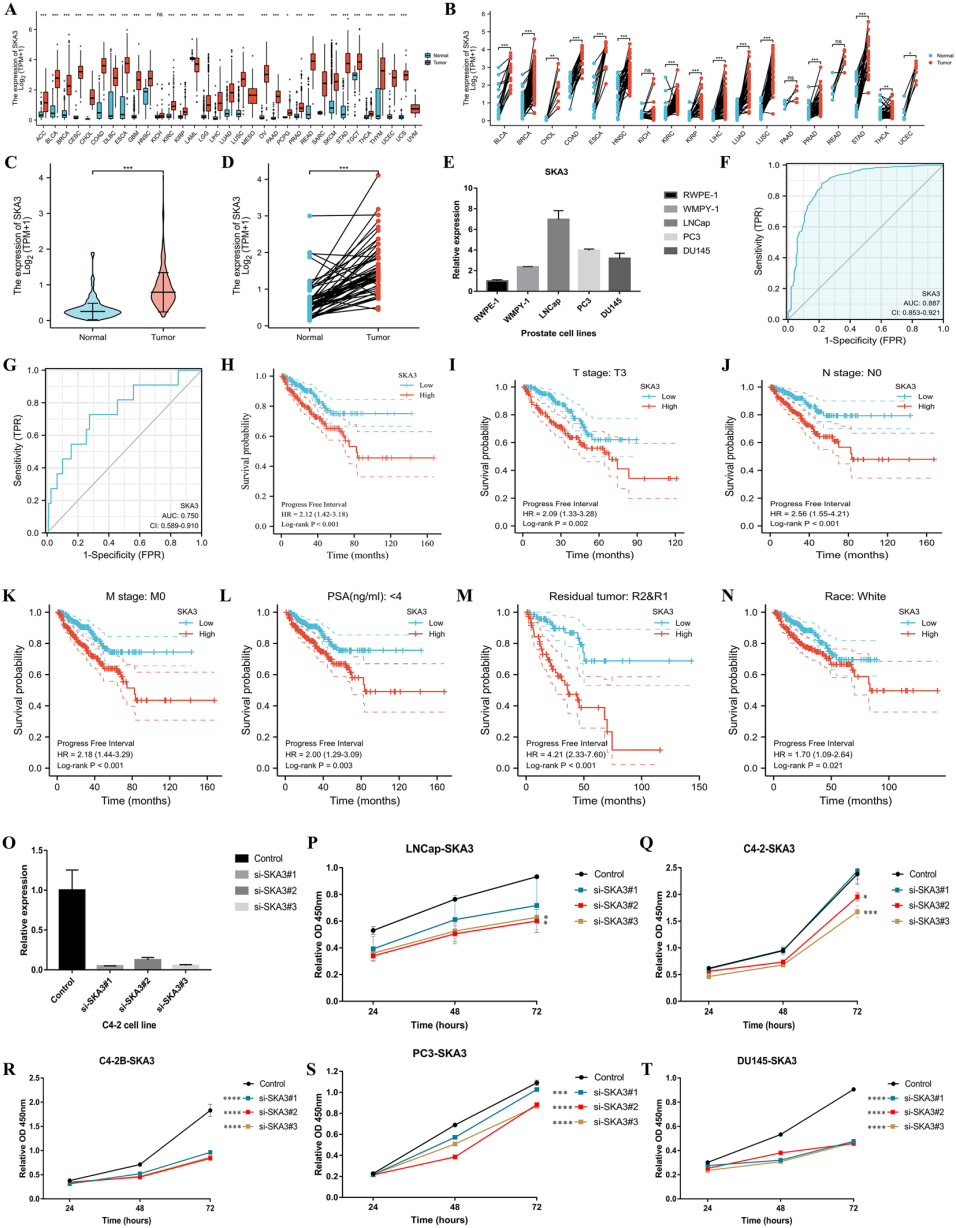
The correlation analysis between SKA3 expression and clinical parameters and the effect of SKA3 expression on the proliferation ability of PCA cells. **A** The differential expressions of SKA3 in pan-cancer level using non-paired samples; **B** the differential expressions of SKA3 in pan-cancer level using paired samples; **C** the differential expressions of SKA3 in PCA using non-paired samples; **D** the differential expressions of SKA3 in PCA using paired samples; **E** Relative expression of SKA3 among prostate normal and tumor cell lines; **F** the ROC curve of SKA3 distinguishing tumor from normal; **G** the ROC curve of SKA3 distinguishing T2–3 from T4; **H** Kaplan–Meier curve showing progress free survival of high and low expression of SKA3; **I** Kaplan–Meier curve showing progress free survival of high and low expression of SKA3 in patients with T3 stage; **J** Kaplan–Meier curve showing progress free survival of high and low expression of SKA3 in patients with N0 stage; **K** Kaplan-Meier curve showing progress free survival of high and low expression of SKA3 in patients with M0 stage; **L** Kaplan–Meier curve showing progress free survival of high and low expression of SKA3 in patients with PSA < 4 ng/ml; **M** Kaplan–Meier curve showing progress free survival of high and low expression of SKA3 in patients with positively residual tumor; **N** Kaplan–Meier curve showing progress free survival of high and low expression of SKA3 in white patients; **O** RT–qPCR results of SKA3 siRNAs; **P** effect of SKA3 siRNAs on LNCap using CCK8 assay; **Q** effect of SKA3 siRNAs on C4-2 using CCK8 assay; **R** effect of SKA3 siRNAs on C4-2B using CCK8 assay; **S** effect of SKA3 siRNAs on PC3 using CCK8 assay; **T** effect of SKA3 siRNAs on DU145 using CCK8 assay. ROC = receiver operating characteristic curve. R = residual tumor. RT–qPCR = Real-time quantitative polymerase chain reaction

**Table 1:** The relationships between SKA3 expression and clinicopathological features in prostate cancer patients in the TCGA database

Characteristic	Low expression of SKA3 (n = 249)	High expression of SKA3 (n = 250)	P value
Age, n (%)			0.080
< = 60	122 (54.5%)	102 (45.5%)	
> 60	127 (46.2%)	148 (53.8%)	
T stage, n (%)			< 0.001
T2	124 (65.6%)	65 (34.4%)	
T3	118 (40.4%)	174 (59.6%)	
T4	2 (18.2%)	9 (81.8%)	
N stage, n (%)			0.036
N0	171 (49.3%)	176 (50.7%)	
N1	28 (35.4%)	51 (64.6%)	
M stage, n (%)			1.000
M0	223 (49%)	232 (51%)	
M1	1 (33.3%)	2 (66.7%)	
Race, n (%)			0.101
Asian	4 (33.3%)	8 (66.7%)	
Black or African American	35 (61.4%)	22 (38.6%)	
White	202 (48.7%)	213 (51.3%)	
Residual tumor, n (%)			0.006
R0	173 (54.9%)	142 (45.1%)	
R1	59 (39.9%)	89 (60.1%)	
R2	2 (40%)	3 (60%)	
Zone of origin, n (%)			0.348
Central Zone	2 (50%)	2 (50%)	
Overlapping / Multiple Zones	50 (39.7%)	76 (60.3%)	
Peripheral Zone	69 (50.4%)	68 (49.6%)	
Transition Zone	3 (37.5%)	5 (62.5%)	
PSA (ng/ml), n (%)			0.102
< 4	214 (51.6%)	201 (48.4%)	
>=4	9 (33.3%)	18 (66.7%)	
Gleason score, n (%)			< 0.001
6	36 (78.3%)	10 (21.7%)	
7	142 (57.5%)	105 (42.5%)	
8	28 (43.8%)	36 (56.2%)	
9	42 (30.4%)	96 (69.6%)	
10	1 (25%)	3 (75%)	
PFI event, n (%)			< 0.001
Alive	218 (53.8%)	187 (46.2%)	
Dead	31 (33%)	63 (67%)	

*SKA3* spindle and kinetochore-associated complex subunit 3; *TCGA* the Cancer Genome Atlas; *PFI* progress free interval

## Supplementary Information

**Additional file 1. **The full text of original manuscript containing figures.

## Data Availability

The datasets presented in this study can be found in online repositories. The names of the repository/repositories and accession number(s) can be found in the article/Additional file 1.
