# Supplementary material for: Spindle and kinetochore-associated complex subunit 3 could serve as a prognostic biomarker for prostate cancer
Source: Exp Hematol Oncol. 2022 Oct 20;11:76. doi: 10.1186/s40164-022-00337-3 (PMC9583514; doi:10.1186/s40164-022-00337-3)
Supplement: Supplementary file 1 — Additional file 1. The full text of original manuscript containing figures. [file 40164_2022_337_MOESM1_ESM.docx]

For readers to better understand our study, wo provided the full-text articles and detailed methods.

**Title:** Spindle and kinetochore-associated complex subunit 3 could serve as a prognostic biomarker for prostate cancer

**Running title**: clinical value of SKA3 in prostate adenocarcinoma

**Authors:**

Dechao Feng, Ph.D.^1, *^, Weizhen Zhu, M.D. ^1, *^, Xu Shi, M.D. ^1, *^, Qiao Xiong, Ph.D.^1^, Dengxiong Li, Ph.D.^1^, Wuran Wei, Ph.D.^1^, Ping Han, Ph.D.^1^, Qiang Wei, M.D. ^1, &^, Lu Yang, Ph.D.^1, &^

1 Department of Urology, Institute of Urology, West China Hospital, Sichuan University, Chengdu 610041, China

* These authors contributed equally to this work.

& Corresponding Author: Department of Urology, Institute of Urology, West China Hospital, Sichuan University, Guoxue Xiang #37, Chengdu, 610041, Sichuan, People's Republic of China.

Tel: +86-28-85422444

Fax: +86-28-85422451

**E-mail:**

Qiang Wei: [weiqiang933@126.com](mailto:weiqiang933@126.com);

Lu Yang: [wycleflue@scu.edu.cn](mailto:wycleflue@scu.edu.cn).

**Abstract**

**Background:** Spindle and kinetochore-associated complex subunit 3 (SKA3) is a microtubule-binding subcomplex of the outer kinetochore which is essential for normal chromosome segregation and cell division. However, little is known about the potential mechanism of SKA3, especially in respect of progression of prostate cancer (PCA).

**Methods:** TCGA, UCSC XENA, STRING, GTEx, Oncomine, and CBioportal databases were used to analyze the expression of SKA3 in PCA patients, and to illuminate the clinical value and possible mechanism of SKA3 in the occurrence and development of PCA. A nomogram was established based on the results of Cox regression analyses, while Harrell's concordance index (C-index) and calibration curve were used to evaluate the nomogram model. RT-qPCR and CCK8 assay were used tested the biological function of SKA3 in vitro.

**Results:** SKA3 was significantly upregulated in patients with PCA, and associated with lower progress free interval (PFI). Moreover, we found that SKA3 mRNA expression was higher in PCA cells than normal cells, and inhibition of SKA3 could obviously decrease the proliferation ability of PCA cells using CCK8 assay. Gene ontology (GO) and Kyoto Encyclopedia of Genes and Genome (KEGG) showed that the genes co-expressed with SKA3 were mainly enriched in homologous recombination, organelle fission, nuclear division, chromosomal region, cell cycle, DNA replication, p53 signaling pathway and so on. The ten hub genes in protein-protein interactions were CDK1, CDC20, BUB1, CCNB1, CCNA2, AURKB, CCNB2, BUB1B, TOP2A, and MAD2L1, which were differentially expressed and associated with PCA progression as well. Gene Set Enrichment Analysis (GSEA) indicated that the highly enriched function and pathways in response to SKA3 upregulation included p53 pathway, DNA replication, FOXM1 pathway, cell cycle, FCERI mediated MAPK activation, MYC active pathway, ATM signaling pathway, steroid hormone biosynthesis, hypoxia, apoptosis, epithelial mesenchymal transition (EMT) and so on. Gene set variation analysis (GSVA) of SKA3 and its 10 hub genes showed that the significant correlation of cancer related pathways included cell cycle, DNA damage, hormone androgen receptor, and EMT. In addition, we found that MEK-inhibitors, i.e., trametinib, selumetinib and RDEA119, may be feasible targeted agents for PCA patients.

**Conclusion:** SKA3 might serve as a prognostic biomarker for patients with PCA. The related pathways, such as activation of the cell cycle, DNA damage, hormone androgen receptors, and inhibition of EMT, still need to be further studied in vivo and in vitro.

**Keywords:** spindle and kinetochore associated complex subunit 3; prostate adenocarcinoma; biomarker; enrichment analysis; targeted therapy.

**Introduction**

Prostate cancer (PCA) has always been recognized as a serious, worldwide public health concern in men, with an estimated almost 1.4 million new cases and 375,000 deaths worldwide [1]. In 2020, PCA is the second most common cancer and the fifth leading cause of death from cancer among men worldwide, and the incidence rate continues to rise in China [1-2]. Epidemiologic studies have established considerable risk factors linked to the development of PCA, such as age, race, family history, and environmental and lifestyle factors [3-4]. However, the conclusive association between such risk factors and PCA development has not been demonstrated. Several studies have developed various risk stratification systems based on clinicopathological parameters, such as the University of California, San Francisco Cancer of the Prostate Risk Assessment (CAPRA) score [5], and the original preoperative and postoperative Kattan nomograms [6-7]. To date, however, previous studies have failed to sufficiently predict prognostic outcome, resulting in overtreatment or undertreatment, thereby leading to tumor progression, complications and an increased risk of cancer-specific survival [8-9].

PCA has been shown to be one of the most heritable cancers, with up to 57% heritable etiology, and consists of manifold frequent and a few rare germline genetic variants [4,10]. It is well-known that the prognosis of PCA varies considerably. Most cases are cured by initial treatment or may be given active surveillance with impunity, while some men suffer from disease occurrence, metastasis and even death [11]. The differences in etiology and clinical behavior can be partially attributed to genetic heterogeneity [12]. Genome-wide association studies (GWAS) identified 76 variants associated with prostate cancer risk, which could explain approximately 30% of the familial risk [10]. Al Olama et al. [13] found 23 additional susceptibility loci, elucidating 33% of the familial risk for this disease in European-ancestry populations in combination with known PCA variants. Subsequently, The Cancer Genome Atlas (TCGA) classified 74% of primary PCA as one of seven subtypes according to specific gene fusions (ERG, ETV1/4, and FLI1) or mutations (SPOP, FOXA1, and IDH1) [14]. In the era of whole genome research, the current detection technology allows us to further explore the mechanism of disease onset and progression at the molecular level. Further risk stratifications combining molecular features with clinicopathologic characteristics play a crucial role in discriminating indolent from aggressive PCA. Thus, the importance of determining the factors related to the progression of PCA is indisputable.

Spindle and kinetochore associated complex subunit 3 (SKA3) is a microtubule-binding subcomplex of the outer kinetochore that is essential for proper chromosome segregation and cell division [15-17]. Knockdown of SKA3 expression activates the spindle assembly checkpoint with loss of sister chromatid cohesion, leading to mitotic arrest during metaphase [18]. It has been proven that the differential expression of SKA3 is related to the disease progression and prognosis of some malignant tumors, such as laryngeal squamous cell carcinoma [18], hepatocellular carcinoma [19], breast cancer [20], and lung adenocarcinoma [21]. In addition, our previous study observed that SKA3 might contribute to worse overall survival, disease-specific survival and progression-free survival for patients with kidney renal papillary cell carcinoma [22]. However, there is little published information about the potential molecular mechanism of carcinogenesis of SKA3 in PCA. In this study, we analyzed the differential expression of SKA3, and explored its relationship with clinicopathological features and prognosis, thereby elucidating the underlying mechanism and possible drug treatments in PCA patients based on various public databases.

**Methods**

**Cell lines and reagents**

Human normal prostate epithelial cell (RWPE-1) was purchased from Zhongqiao Xinzhou (<https://www.zqxzbio.com/Index/p_more/pid/779.html>) and cultured in matched keratinocyte complete medium. Human normal prostate stromal cell (WMPY-1) was purchased and authenticated from cell bank (Chinese Academy of Sciences, Shanghai, China) and cultured in DMEM (Invitrogen, CA, United States) with 10% fetal bovine serum (FBS) (CxCell BIO, Uruguay). Human PCA cell lines, LNCap, PC3 and DU145, were purchased and authenticated from cell bank (Chinese Academy of Sciences, Shanghai, China). Human PCA cell lines, C4-2 and C4-2B were purchased from BNCC (<https://www.bncc.org.cn/>). LNCap, C4-2, C4-2B and DU145 were cultured in RPMI medium 1640 (Gibco) with 10% FBS and 5% penicillin-streptomycin solution. PC3 was cultured in DMEM/F12 (Gibco) with 10% FBS and 5% penicillin-streptomycin solution. All cells were incubated in 5% CO2 incubator at 37℃ and were tested for mycoplasma free via a mycoplasma detection kit (Thermo Fisher Scientific, United States).

**Data collections**

Our study has been registered in the ISRCTN registry (No. ISRCTN11560295). We obtained PCA data (HTseq-fregments per kilobase per million (FPKM)) from the cancer genome atlas (TCGA) and Genotype–Tissue Expression (GTEx). Besides, the data were processed through Toil [23]. The prognostic data were obtained from the previous study [24]. Data in FPKM format were converted into transcripts per million reads, and log2 conversion was performed for all data in standardization.

**Analysis of differential expression and clinical value**

The collected data were used to assess the differential expression in pan cancer and PCA, classified as paired and unpaired samples. Oncomine (<https://www.oncomine.org/>) was used to assess the RNA levels of SKA3 between normal and tumor tissues [25]. Besides, the data were also used to calculate diagnostic value using receiver operating characteristic (ROC) curves and survival values. We used PFI as a prognostic factor, which was defined as the period during and after treatment in which a participant was living with a disease that did not worsen. Typically, it is the period from date of diagnosis until 1) locoregional or systemic recurrence, 2) second malignancy, or 3) death from any cause; late deaths not related to cancer or its treatment are excluded [24].

**Analysis of CBioportal**

The cBioPortal for Cancer Genomics (http://cbioportal.org) provides a web resource for exploring, visualizing, and analyzing multidimensional cancer genomics data [26-27]. We used cBioPortal to explore the association between SKA3 mRNA expression (RNA Seq V2 RSEM) and hypoxia-related parameters. Hypoxia-related genes were obtained from a previous study [28], and the hypoxia-related scores included the Buffa, Ragnum, and Winter hypoxia scores.

**Biological functional analysis**

The coexpressed genes of SKA3 were identified with Pearson correlation coefficients (|r| >0.4 and P<0.001). Gene ontology (GO) and Kyoto Encyclopedia of Genes and Genome (KEGG) analyses were performed on coexpressed genes with the package R “clusterProfiler” to explore possible biological functions and signaling pathways affected by SKA3 [29]. GO analysis included biological process (BP), cell composition (CC) and molecular function (MF) (P<0.05 was statistically significant). The TCGA gene dataset was analyzed by gene set enrichment analysis (GSEA). The threshold of significant enrichment was false discovery rate (FDR)<0.25 and p.adjust < 0.05. A protein-protein interaction (PPI) network of SKA3 coexpressed genes was identified through the STRING database (<https://www.string-db.org/>) [30], and the value of high confidence was set as 0.7. The obtained PPI network was imported into Cytoscape 3.7.2 software [31], the first 10 genes were defined as hub genes using the CytoHubba plug-in ranked by degree, and relevant analysis was conducted.

**Analysis of GSCALite**

Gene Set Cancer Analysis (GSCALite) [32] is a user-friendly web server for analyzing a set of genes in cancers with the following functional modules: (i) Differential expression in tumor versus normal, and the survival analysis; (ii) Genomic variations and their survival analysis; (iii) Gene expression associated cancer pathway activity; (iv) miRNA regulatory network for genes; (v) Drug sensitivity for genes; (vi) Normal tissue expression and eQTL for genes. We used the GSCALite database to further analyze mutation information, immune infiltration, and gene set variation analysis (GSVA) of SKA3 and its coexpressed genes [32]. Besides, we also explored the Genomics of Drug Sensitivity in Cancer (GDSC) and the Cancer Therapeutics Response Portal (CTRP) drug sensitivity and expression correlation of the top 10 hub coexpressed genes and SKA3 through GSCALite [32].

**Real-time quantitative polymerase chain reaction (RT-qPCR)**

Total RNA from prostate cells were extracted using the animal total RNA isolation kit (www. foregene.com), and cDNA was synthesized using the iScript advanced cDNA synthesis kit (BioRad). RT-qPCR analysis was performed using the real time PCR easy^TM^-SYBR Green I (FOREGENE, Cat. No. QP-01011/01012/01013/01014) according to the manufacturer’s instructions. Glyceraldehyde-3-phosphate dehydrogenase (GAPDH) was used as an internal control. GAPDH: 5’- CTGGGCTACACTGAGCACC-3’ (forward) and 5’-TCC AAGTGGTCGTTGAGGGCAATG-3’ (reverse); SKA3: 5’- TACACGAGCAAGAAGCCATTAAC-3’ (forward) and 5’- GGATACGATGTACCGCTCAAGT-3’ (reverse).

**Small interfering RNA (siRNA) of SKA3 and cell proliferation**

siRNA was designed by HIPPOBIO ([www.hippobiotec.com](http://www.hippobiotec.com)). SKA3 si-1sense: 5’-GCGACUUUGAAGAUUAUCCTT-3’; SKA3 si-1 antisense: 5’-CGAUAAUCUUCAAAGUCGCTT-3’. SKA3 si-2 sense: 5’-GUUCAGACUCUAAAGGAUGTT-3’; SKA3 si-2 antisense: 5’-CAUCCUUUAGAGUCUGAACTT-3’; SKA3 si-3 sense: 5’-GAUCGUACUUCGUUGGUUUTT-3’; SK3 si-3 antisense: 5’-AAACCAACGAAGUACGAUCTT-3'. Prostate cells were transfected using lipofectamine 2000 (Invitrogen) according to the instructions. RT-qPCR was used to determine the effective siRNA of SKA3. Through transfection of SKA3 siRNAs, the effect of SKA3 on the proliferation of five PCA cell lines was analyzed by cell counting kit-8 (CCK8) assay at 24h, 48h and 72h.

**Statistical analysis**

R 3.6.3 software and its suitable packages (“ggplot2”,” pROC”,” survival”, “survminer”, “clusterProfiler”, and “rms”) were used for statistical analysis. The chi-square test was used to assess differences between categorical variables, and t tests or paired sample t tests were used for continuous variables. The Wilcoxon rank sum test was used if the Shapiro-Wilk normality test was significant. The survival analysis was conducted through Kaplan-Meier curves and log-rank tests. A nomogram was established based on the results of Cox regression analyses, while Harrell's concordance index (C-index) and calibration curve were used to evaluate the nomogram model. All the statistical tests mentioned above are two-sided. P-values of <0.05 were considered statistically significant. Significant marks were as follows: ns, p≥0.05; *, p< 0.05; **, p<0.01; ***, p<0.001.

**Results**

**Basic clinicopathological features and clinical correlation analysis**

A total of 499 PCA samples and 52 tumor-adjacent tissues in the TCGA database were used in this study. A total of 249 patients presented low expression of SKA3 mRNA, while 250 patients had high SKA3 mRNA expression. Patients were diagnosed by core needle biopsy or transurethral resection. The relationships between SKA3 expression and clinicopathological features in PCA patients in the UCSC XENA database are summarized in Table 1.

In the pan-cancer analysis of non-paired samples and paired samples, SKA3 mRNA expression was elevated in most cancers compared to normal tissues (Fig. 1A-B). In unpaired samples, SKA3 expression was higher in the tumor group than in the normal group; the median difference between the two groups was 0.505 (0.448-0.562), and the difference was statistically significant (P <0.001) (Fig. 1C). In paired samples, SKA3 expression was also higher in the tumor group when compared to normal group; the median difference between the two groups was 0.833(0.685 - 1.039), and the difference is statistically significant (P <0.001) (Fig. 1D). Similarly, SKA3 was overexpressed in PCA patients in the Oncomine database, with a fold change of 2.929 and a p value of 2.22E-11 (Fig. 1E). Moreover, RT-qPCR also detected that SKA3 was higher expressed in PCA cell lines than that in normal prostate cell lines (Fig. 1F). We observed that kinetochore proteins NUF2 and CENPA, mitosis regulatory genes TPX2, CDK1, MELK, NCAPG, CDCA5, histone chaperone protein ASF1B, and microtubule motor-related protein KIF5A were positively associated with the expression of SKA3. (Fig. 1G). The area under the curve of ROC were between 0.5 and 1. The closer AUC (AUC) to 1, the better the diagnostic effect. When AUC was 0.5 to 0.7, the accuracy was low; when AUC was 0.7 to 0.9, the accuracy was certain; when the AUC was higher than 0.9, the accuracy was high. The area under the curve (AUC) was 0.887 (95%CI: 0.853-0.921), indicating certain diagnostic accuracy of SKA3 distinguishing tumor for normal patients (Fig. 1H). In the subgroup analysis of T2-3 versus T4, the AUC was 0.750 (95%CI: 0.589-0.910) (Fig. 1I).

We observed a significantly increasing trend in the mRNA expression of SKA3 with the upgrade of T stage (Fig. 1J) and N stage (Fig. 1K), the existence of residual tumor (Fig. 1L), and the increase of Gleason score (Fig. 1M). PCA patients with high SKA3 expression had a worse PFI than those with low expression (P<0.001, Fig. 2A). In addition, patients expressing higher SKA3 experienced significantly shorter PFI when compared to their counterparts in the subgroup analysis of T3 stage, N0 stage, M0 stage, PSA<4 ng/ml, positive residual tumor, and white (this meant white demographic) (Fig. 2B-G).

**Gene enrichment analysis**

GO and KEGG analyses were conducted to uncover the potential function and pathways of SKA3 in PCA. The results showed that SKA3 coexpressed genes were principally involved in homologous recombination, organelle fission, nuclear division, chromosomal region, cell cycle, DNA replication, p53 signaling pathway and so on (Fig. 2H-I). Cell cycle and mitosis regulatory genes CDK1, CDC20, BUB1, CCNB1, CCNA2, AURKB, CCNB2, BUB1B, TOP2A, and MAD2L1 were the ten hub genes of SKA3 (Fig. 2J), which were differentially expressed and associated with PCA progression (Fig. 3A-L). In addition, GSEA results indicated that E2F pathway (Fig. 4A-B), cell cycle (Fig. 4B), homologous recombination (Fig.4B), and the transcription of androgen receptor (AR) (Fig. 4E-F) were highly enriched upon the SKA3 overexpression. The GSVA analysis was used to score the PCA patients, and the tumor patents showed higher GSVA score than normal patients (Fig. 4G), and the correlations between GSVA score and pathways in the PCA patients was analyzed (Fig. 4H-I). Similar to the above results, the cell cycle, DNA damage and hormone AR were positively associated with SKA3 and its hub genes with statistical significance through the GSVA analysis (Fig. 4H-I).

**Analysis of GSCALite, and CBioportal**

We analyzed the mutations of SKA3 and its 10 hub genes, including single nucleotide variation (SNV), copy number variation (CNV), and methylation (supplementary figure 1). Besides, the relationship of mutations with survival of PCA was conducted as well (supplementary figure 1). We observed that patients with gene set mutations had a higher risk of PFI than those with wild-type SNVs (HR: 16.67, p=1.82e-5, supplementary figure 1E). The correlation of CNV with mRNA expression was significant in terms of SKA3, MAD2L1, CDK1, CDC20, and CCNB1 (supplementary figure 1I). In addition, we found a negative correlation between methylation and mRNA expression for SKA3 and its hub genes (supplementary figure 1N).

The cut-off used to assess the correlation of SKA3 with immune-infiltrating cells was + 0.3. At the single-gene level, SKA3 mRNA expression was positively correlated with monocytes, macrophages, and dendritic cells (DCs), and negatively related to natural killer T cells (NKTs) and natural killer cells (NKs) (Fig. 5A). However, the mutations (CNV and methylation) of SKA3 were not associated with immune infiltration (Fig. 5B-C). Compared to wild-type samples, DCs, monocytes, macrophages, nTregs, iTregs and B cells were enriched in higher amplification of the gene set, and DCs, monocytes, macrophages, infiltration scores and neutrophil were enriched in deletion of the gene set (Fig. 5D). With the help of GSCALite, we found that MEK-inhibitors, i.e., trametinib, selumetinib and RDEA119, may be feasible targeted agents in PCA patients with high expression of SKA3 and its coexpressed genes (Fig. 5F-G).

The 413 patients with complete clinical information from the TCGA dataset were used to establish a nomogram predicting 1-, 3-, and 5-year PFI based on the stepwise Cox regression model (Fig. 6A). A calibration plot showed that the nomogram had a certain predictive ability for predicting PFI in PCA patients (Fig. 6B). In addition, the analysis of mutual exclusivity showed that SKA3 and hypoxia-related genes [28] functioned together in tumor hypoxia through CBioportal (Fig. 6C). The hypoxia scores (Buffa, Ragnum, and Winter) were significantly higher in the SKA3-altered group than those in the unaltered group (Fig. 6D-F).

**Cell proliferation**

We found that SKA3 expression was downregulated after transfection of the three siRNAs using the RT-qPCR assay (Fig. 7A). Moreover, these siRNAs could significantly reduce the ability of PCA cells to proliferate (Fig. 7B-F), especially for the cells with high levels of malignancy, such as C4-2B, PC3, and DU145, which was consistent with the above clinical correlations of SKA3.

**Discussion**

The prognosis of PCA presents a widely variable clinical behavior. The 5-year survival rate for patients with metastatic PCA is approximately 28%, while this rate is nearly 100% for those with localized tumors [33]. Thus, metastasis is a predominant risk factor leading to death in PCA patients. Although the mortality rate of PCA has been reduced by approximately 40% in the past two decades, questions about the prevention of prostate cancer progression and necessary interventions before prostate cancer metastasizes to other organs remain poorly understood [33]. More recent attention has focused on the provision of molecular and genetic profiles to define cancer subtypes and further provides insights to precisely targeted treatments since the completion of the TCGA project. However, many of them are exploratory in nature due to the complexity and multidimensionality of huge genomic data. In PCA, the most frequent alterations are fusions of androgen-regulated promoters with ERG and other members of the ETS family of transcription factors, in which the TMPRSS2-ERG fusion is the most common molecular alteration [14, 34]. However, the fusion-bearing difference does not lead to significant survival benefits to PCA patients following prostatectomy compared to those without [35]. Thus, there is a growing body of literature that recognizes the importance of understanding the biology of genetic variants, which could indeed predict response to certain therapies, or identify those who are at increased risk of treatment related toxicities [10]. Previous studies have reported that SKA3 might contribute to the progression of several malignancies, such as laryngeal squamous cell carcinoma [18], hepatocellular carcinoma [19], breast cancer [20], lung adenocarcinoma [21], and kidney renal papillary cell carcinoma [22]. Consistent with the previous studies [18-22], in our study, we observed that SKA3 mRNA expression was significantly increased in most cancers than normal tissues. However, the correlation between SKA3 and PCA has not been reported to date. In our study, we found that SKA3 mRNA expression was higher in PCA cells than normal cells, and inhibition of SKA3 could obviously decrease the proliferation ability of PCA cells. In addition, patients with higher mRNA expression of SKA3 had a higher risk of progression than those with lower mRNA expression of SKA3. Moreover, we observed that patients with T3 stage, N0 stage, M0 stage, PSA<4 ng/ml, positive residual tumor, or white patients were prone to progress if they highly expressed SKA3 mRNA. These results showed that higher SKA3 expression could promote the occurrence and progression of PCA, and has a good clinical correlation. Moreover, we proposed the dosage effect of SKA3 carcinogenesis based on the above subgroup analyses. Thus, SKA3 might involve in the progression of PCA and serve as a prognostic biomarker for PCA patients.

In this study, we observed that many cell cycle-related genes, such as kinetochore proteins NUF2 and CENPA, mitosis regulatory genes TPX2, CDK1, MELK, NCAPG, CDCA5, histone chaperone protein ASF1B, and microtubule motor-related protein KIF5A, were highly associated with the expression and function of SKA3. The roles of these genes on the tumorigenesis and progression of PCA were presented as follows. Low rates of chromosome missegregation can promote tumor development, whereas higher levels might promote cell death and suppress tumorigenesis; TPX2 plays a critical role in chromosome segregation machinery during mitosis [36]. Previous studies indicated that overexpression of TPX2 contributed to progression of PCA [37], and TPX2 silencing led to deregulation of CDK1, cyclin B, securin, separase, and aurora A proteins [36]. In addition, TPX2 mediated PCA EMT through CDK1 regulated phosphorylation of ERK/GSK3β/SNAIL pathway [37]. NUF2 was associated with the poor prognosis of PCA [38, 39], CENPA could serve as a transcriptional regulator that modulated expression of critical proliferation, cell-cycle, and centromere/kinetochore genes [40, 41]. MELK was upregulated in high-grade PCA, and BUB1B promoted proliferation of PCA via transcriptional regulation of MELK [42, 43]. Besides, Regulation of NCAPG by miR-99a-3p (passenger strand) inhibits cancer cell aggressiveness and is involved in castration-resistant PCA [44]. CDCA5 promoted the progression of PCA by affecting the ERK signaling pathway, and downregulation of CDCA5 could inhibit cell proliferation, migration, and invasion, and induce apoptosis of PCA cells [45, 46]. Moreover, SPOP promoted CDCA5 degradation to regulate PCA progression via the AKT pathway [47]. Knockdown of ASF1B induced cell apoptosis via repressing PI3K/Akt pathway in PCA, and expression of ASF1B and MCL1 were independent prognostic factors of biochemical recurrence [48, 49]. Compared with normal prostate, the mRNA and protein expressions of KIF4A were up-regulated in PCA, and the elevated KIF4A level also predicted independently a poor biochemical recurrence-free and disease-free survival of PCA patients [50]. Furthermore, targeting the KIF4A/ androgen receptor (AR) axis could reverse endocrine therapy resistance in castration-resistant PCA [51]. The checkpoint catalyst was substrate assisted and promotes the assembly of mitotic checkpoint complex through spatially and temporally coordinated conformational changes in both MAD2 and CDC20 [52]. Overexpression of CDC20 was highly associated with high Gleason score and biochemical recurrence in patients with clinically localized PCA after laparoscopic radical prostatectomy [53]. In addition, CDC20 could drive PCA progression via stabilization of β-catenin in cancer stem-like cells [54]. BUB1, CCNA2, CCNB1, CDK1, MAD2L1, and PLK1 might play a critical role in interaction with CDC20 [55]. In this study, we observed that SKA3 was highly associated with the above genes. Almost all of these played an important role in the process of cell cycle, and it is noteworthy that half of the SKA3-related genes, such as TPX2, CDK1, MELK, NCAPG, CDCA5, CDC20, BUB1, NUF2, and CENPA, were closely associated with mitotic checkpoint or kinetochore structure, which indicated that enrichment of mitotic checkpoint genes may contribute to more faithful mitosis, and therefore tumor progression. Besides, all SKA3-hub genes (CDK1, CDC20, BUB1, CCNB1, CCNA2, AURKB, CCNB2, BUB1B, TOP2A, and MAD2L1) were significantly expressed higher in tumor samples than normal tissues, and were associated with progression of PCA. Thus, SKA3 might involve in the PCA progression through interaction with these genes, and methylation of these genes might suppress the PCA progression through downregulating their mRNA expression. Furthermore, we also detected that SKA3 mRNA expression was significantly negative with the number of NKTs and NKs, which indicated that the ability of NKTs and NKs to kill tumor cells was weakened and promoted the PCA progression.

AR is a transcription factor responsible for mediating the effects of androgens on target tissues, and plays a pivotal role in the development and progression of PCA [56]. We found that SKA3 together with its coexpressed genes might contribute to upregulation of hormone AR, leading to the progression of PCA under the synergistic effect of the cell cycle and DNA damage. In addition, the reversal of EMT might promote this process. Despite androgen deprivation therapy, low levels of circulating androgens persist and AR can be transactivated in the low-androgen environment through various pathways and genetic aberrations, such as hypoxia resulting from androgen deprivation as a result of castration [56-59]. A strong relationship between hypoxia and tumors has been reported in prior studies [60-61]. Hypoxia leads to a series of biological changes that induce tumorigenesis and is associated with resistance to chemotherapy, radiation therapy, drug therapy and immunotherapy [62]. PCA is largely driven by abnormal androgen signals and tumor hypoxia [56]. We found that altered SKA3 was positively associated with the three hypoxia-related scores, namely Buffa, Ragnum, and Winter hypoxia scores. Furthermore, the analysis of mutual exclusivity showed that SKA3 and hypoxia-related genes identified by the previous study [28] together contribute to PCA hypoxia. Thus, SKA3 may be involved in tumor progression through hypoxia-related pathways or AR-related pathways under the hypoxic condition. The GSEA results indicated that SKA3 might be involved in the regulation of AR through related pathways, such as steroid hormone biosynthesis, and activated PKN1 stimulates the transcription of the androgen receptor regulated genes KLK2 and KLK3. Other SKA3-related pathways, such as regulation of TP53 activity, MYC activation pathway, signaling by NOTCH, E2F targets, Glucuronidation, AURORA-B pathway, DNA replication, FOXM1 pathway, FCERI mediated MAPK activation, homologous recombination, E2F pathway, FCERI mediated NF-KB activation, cell cycle, ATM signaling pathway, reproduction, and TP53 regulates transcription of cell cycle genes, also provide directions for the mechanism of tumor progression in the future.

In our study, we found that MEK-inhibitors, i.e., trametinib, selumetinib and RDEA119), may be sensitive to PCA patients with high expression of SKA3 and its coexpressed genes. Nickols et al. proposed that pharmacologic targeting of the MEK/ERK pathway may be a viable treatment strategy for patients with refractory metastatic prostate cancer [63]. Currently, studies about trametinib and selumetinib are ongoing [63-64]. In addition, for those patients who choose nonsurgical treatment, our predictive model may enable doctors to inform patients of the probability of disease progression, thereby helping patients make medical decisions.

We did admit the following limitation in our study. (1) Validation using clinical samples is absent; (2) the nomogram model needs external validation and modification, and it can be used as a reference currently; (3) further verification of SKA3 function at the cellular level is needed; (4) a previous study indicated that overexpression of SKA3 caused a significant increase in cells and colony formation, but led to decrease in cell migration and invasiveness [65]. This result may be related to his choice of mouse model mimicking human neuroendocrine prostate cancer.

**Conclusion**

SKA3 might serve as a prognostic biomarker for patients with PCA. The related pathways, such as activation of the cell cycle, DNA damage, hormone androgen receptors, and inhibition of EMT, still need to be further studied in vivo and in vitro.

**Declarations**

**Ethical Approval and Consent to participate**

The authors are accountable for all aspects of the work in ensuring that questions related to the accuracy or integrity of any part of the work are appropriately investigated and resolved.

**Consent for publication**

Not applicable.

**Funding**

This program was supported by the National Natural Science Foundation of China (Grant Nos. 81974099, 82170785, 81974098, 82170784), programs from Science and Technology Department of Sichuan Province (Grant Nos. 21GJHZ0246), Young Investigator Award of Sichuan University 2017 (Grant No. 2017SCU04A17), Technology Innovation Research and Development Project of Chengdu Science and Technology Bureau (2019-YF05-00296-SN), Sichuan University--Panzhihua science and technology cooperation special fund (2020CDPZH-4). The funders had no role in the study design, data collection or analysis, preparation of the manuscript, or the decision to publish.

**Availability of supporting data**

The datasets presented in this study can be found in online repositories. The names of the repository/repositories and accession number(s) can be found in the article/supplementary material.

**Competing interests**

The authors have no conflicts of interest to declare.

**Authors' contributions**

DCF proposed the project, conducted data analysis, interpreted the data, and wrote the manuscript; WZZ, XS, DXL, and QX conducted data analysis, interpreted the data; QW and LY, supervised the project, and interpreted the data. All authors reviewed and edited the manuscript.

**Acknowledgements**

The results showed here are in whole or part based upon data generated by the Genotype-Tissue Expression (GTEx) Program (https://commonfund.nih.gov/GTEx/) and TCGA Research Network (https://www.cancer.gov/tcga).

**References**

1. Sung H, Ferlay J, Siegel RL, et al. Global Cancer Statistics 2020: GLOBOCAN Estimates of Incidence and Mortality Worldwide for 36 Cancers in 185 Countries. CA Cancer J Clin. 2021;71(3):209-249.

2. Culp MB, Soerjomataram I, Efstathiou JA, et al. Recent global patterns in prostate cancer incidence and mortality rates. Eur Urol. 2020; 77 (1):38-52.

3. Zuniga KB, Chan JM, Ryan CJ, et al. Diet and lifestyle considerations for patients with prostate cancer. Urol Oncol. 2020;38(3):105-117.

4. Vigneswaran HT, Jagai JS, Greenwald DT, et al. Association between environmental quality and prostate cancer stage at diagnosis. Prostate Cancer Prostatic Dis. 2021. doi: 10.1038/s41391-021-00370-z. Epub ahead of print.

5. Cooperberg MR, Broering JM, Carroll PR. Risk assessment for prostate cancer metastasis and mortality at the time of diagnosis. J Natl Cancer Inst. 2009;101(12):878-87.

6. Graefen M, Karakiewicz PI, Cagiannos I, et al. A validation of two preoperative nomograms predicting recurrence following radical prostatectomy in a cohort of European men. Urol Oncol. 2002;7(4):141–146.

7. Graefen M, Karakiewicz PI, Cagiannos I, et al. Validation study of the accuracy of a postoperative nomogram for recurrence after radical prostatectomy for localized prostate cancer. J Clin Oncol. 2002;20(4):951–956.

8. Schröder FH, Hugosson J, Roobol MJ, et al. Prostate-Cancer mortality at 11 years of follow-up. N Engl J Med. 2012;366(11):981–990.

9. Aizer AA, Chen MH, Hattangadi J, et al. Initial management of prostate-specific antigen-detected, low-risk prostate cancer and the risk of death from prostate cancer. BJU Int. 2014;113(1):43–50.

10. Eeles R, Goh C, Castro E, et al. The genetic epidemiology of prostate cancer and its clinical implications. Nat Rev Urol. 2014;11(1):18-31.

11. Klotz L. Active surveillance in intermediate-risk prostate cancer. BJU Int. 2020;125(3):346-354.

12. Eeles R, Goh C, Castro E, et al. The genetic epidemiology of prostate cancer and its clinical implications. Nat Rev Urol. 2014;11(1):18-31.

13. Al Olama AA, Kote-Jarai Z, Berndt SI, et al. A meta-analysis of 87,040 individuals identifies 23 new susceptibility loci for prostate cancer. Nat Genet. 2014;46(10):1103-9.

14. Cancer Genome Atlas Research Network. The Molecular Taxonomy of Primary Prostate Cancer. Cell. 2015;163(4):1011-25.

15. Welburn JP, Grishchuk EL, Backer CB, et al. The human kinetochore Ska1 complex facilitates microtubule depolymerization-coupled motility. Dev Cell. 2009;16(3):374-85.

16. Gaitanos TN, Santamaria A, Jeyaprakash AA, et al. Stable kinetochore-microtubule interactions depend on the Ska complex and its new component Ska3/C13Orf3. EMBO J. 2009;28(10):1442-52.

17. Schmidt JC, Arthanari H, Boeszoermenyi A, et al. The kinetochore-bound Ska1 complex tracks depolymerizing microtubules and binds to curved protofilaments. Dev Cell. 2012;23(5):968-80.

18. Gao W, Zhang Y, Luo H, et al. Targeting SKA3 suppresses the proliferation and chemoresistance of laryngeal squamous cell carcinoma via impairing PLK1-AKT axis-mediated glycolysis. Cell Death Dis. 2020;11(10):919.

19. Hou Y, Wang Z, Huang S, et al. SKA3 Promotes tumor growth by regulating CDK2/P53 phosphorylation in hepatocellular carcinoma. Cell Death Dis. 2019;10(12):929.

20. Zhang J, Liu Y, Pu S, et al. Spindle and kinetochore‑associated complex subunit 3 accelerates breast cancer cell proliferation and invasion through the regulation of Akt/Wnt/β-catenin signaling. Breast Cancer Res Treat. 2021;186(1):247-258.

21. Hu DD, Chen HL, Lou LM, et al. SKA3 promotes lung adenocarcinoma metastasis through the EGFR-PI3K-Akt axis. Biosci Rep. 2020;40(2):BSR20194335.

22. Feng D, Zhang F, Liu L, et al. SKA3 Serves as a Biomarker for Poor Prognosis in Kidney Renal Papillary Cell Carcinoma. Int J Gen Med. 2021; 14:8591-8602.

23. Vivian J, Rao A A, Nothaft F A, et al. Toil enables reproducible, open source, big biomedical data analyses. Nature biotechnology, 2017, 35(4): 314-316.

24. Liu J, Lichtenberg T, Hoadley KA, et al. An Integrated TCGA Pan-Cancer Clinical Data Resource to Drive High-Quality Survival Outcome Analytics. Cell. 2018;173(2):400-416.e11.

25. Grasso CS, Wu YM, Robinson DR, et al. The mutational landscape of lethal castration-resistant prostate cancer. Nature. 2012;487(7406):239-43.

26. Cerami E, Gao J, Dogrusoz U, et al. The cBio cancer genomics portal: an open platform for exploring multidimensional cancer genomics data. Cancer Discov. 2012;2(5):401-4.

27. Gao J, Aksoy BA, Dogrusoz U, et al. Integrative analysis of complex cancer genomics and clinical profiles using the cBioPortal. Sci Signal. 2013;6(269):pl1.

28. Bhandari V, Hoey C, Liu LY, et al. Molecular landmarks of tumor hypoxia across cancer types. Nat Genet. 2019;51(2):308-318.

29. Yu G, Wang L G, Han Y, et al. clusterProfiler: an R package for comparing biological themes among gene clusters. Omics: a journal of integrative biology. 2012, 16(5): 284-287.

30. Szklarczyk D, Gable AL, Nastou KC, et al. The STRING database in 2021: customizable protein-protein networks, and functional characterization of user-uploaded gene/measurement sets. Nucleic Acids Res. 2021;49(D1):D605-D612.

31. Shannon P, Markiel A, Ozier O, et al. Cytoscape: a software environment for integrated models of biomolecular interaction networks. Genome Res. 2003;13(11):2498-504.

32. Liu CJ, Hu FF, Xia MX, et al. GSCALite: a web server for gene set cancer analysis. Bioinformatics. 2018;34(21):3771-3772.

33. Chen Q, Yao YT, Xu H, et al. SPOCK1 promotes tumor growth and metastasis in human prostate cancer. Drug Des Devel Ther. 2016;10:2311-2321.

34. Tomlins SA, Rhodes DR, Perner S, et al. Recurrent fusion of TMPRSS2 and ETS transcription factor genes in prostate cancer. Science. 2005;310(5748):644-8.

35. Gopalan A, Leversha MA, Satagopan JM, et al. TMPRSS2-ERG gene fusion is not associated with outcome in patients treated by prostatectomy. Cancer Res. 2009 15;69(4):1400–6

36. Pan HW, Su HH, Hsu CW, et al. Targeted TPX2 increases chromosome missegregation and suppresses tumor cell growth in human prostate cancer. Onco Targets Ther. 2017; 10:3531-3543.

37. Zou J, Huang RY, Jiang FN, Chen DX, Wang C, Han ZD, Liang YX, Zhong WD. Overexpression of TPX2 is associated with progression and prognosis of prostate cancer. Oncol Lett. 2018;16(3):2823-2832.

38. Zhang B, Zhang M, Li Q, et al. TPX2 mediates prostate cancer epithelial-mesenchymal transition through CDK1 regulated phosphorylation of ERK/GSK3β/SNAIL pathway. Biochem Biophys Res Commun. 2021; 546:1-6.

39. Pezeshki S, Hashemi P, Salimi A, et al. Evaluation of NUF2 and GMNN Expression in Prostate Cancer: Potential Biomarkers for Prostate Cancer Screening. Rep Biochem Mol Biol. 2021;10(2):224-232.

40. Chen X, Ma Q, Liu Y, et al. Increased expression of CELSR3 indicates a poor prognostic factor for Prostate Cancer. J Cancer. 2021;12(4):1115-1124.

41. Saha AK, Contreras-Galindo R, Niknafs YS, et al. The role of the histone H3 variant CENPA in prostate cancer. J Biol Chem. 2020;295(25):8537-8549.

42. Kuner R, Fälth M, Pressinotti NC, et al. The maternal embryonic leucine zipper kinase (MELK) is upregulated in high-grade prostate cancer. J Mol Med (Berl). 2013;91(2):237-48.

43. Tian JH, Mu LJ, Wang MY, Zeng J, Long QZ, Guan B, Wang W, Jiang YM, Bai XJ, Du YF. BUB1B Promotes Proliferation of Prostate Cancer via Transcriptional Regulation of MELK. Anticancer Agents Med Chem. 2020;20(9):1140-1146.

44. Arai T, Okato A, Yamada Y, et al. Regulation of NCAPG by miR-99a-3p (passenger strand) inhibits cancer cell aggressiveness and is involved in CRPC. Cancer Med. 2018;7(5):1988-2002.

45. Ji J, Shen T, Li Y, et al. CDCA5 promotes the progression of prostate cancer by affecting the ERK signalling pathway. Oncol Rep. 2021;45(3):921-932.

46. Chong Y, Xue L. Downregulation of CDCA5 Can Inhibit Cell Proliferation, Migration, and Invasion, and Induce Apoptosis of Prostate Cancer Cells. Crit Rev Eukaryot Gene Expr. 2021;31(1):29-40.

47. Luo Z, Wang J, Zhu Y, et al. SPOP promotes CDCA5 degradation to regulate prostate cancer progression via the AKT pathway. Neoplasia. 2021;23(10):1037-1047.

48. Han G, Zhang X, Liu P, et al. Knockdown of anti-silencing function 1B histone chaperone induces cell apoptosis via repressing PI3K/Akt pathway in prostate cancer. Int J Oncol. 2018;53(5):2056-2066.

49. Carrion A, Ingelmo-Torres M, Lozano JJ, et al. Prognostic classifier for predicting biochemical recurrence in localized prostate cancer patients after radical prostatectomy. Urol Oncol. 2021;39(8):493.e17-493.e25.

50. Chen J, Li M, Fang S, et al. KIF4A: A potential biomarker for prediction and prognostic of prostate cancer. Clin Invest Med. 2020;43(3):E49-59.

51. Cao Q, Song Z, Ruan H, et al. Targeting the KIF4A/AR Axis to Reverse Endocrine Therapy Resistance in Castration-resistant Prostate Cancer. Clin Cancer Res. 2020 ;26(6):1516-1528.

52. Piano V, Alex A, Stege P, et al. CDC20 assists its catalytic incorporation in the mitotic checkpoint complex. Science. 2021;371(6524):67-71.

53. Mao Y, Li K, Lu L, et al. Overexpression of Cdc20 in clinically localized prostate cancer: Relation to high Gleason score and biochemical recurrence after laparoscopic radical prostatectomy. Cancer Biomark. 2016;16(3):351-8.

54. Zhang Q, Huang H, Liu A, et al. Cell division cycle 20 (CDC20) drives prostate cancer progression via stabilization of β-catenin in cancer stem-like cells. EBioMedicine. 2019 ;42:397-407.

55. Wu F, Sun Y, Chen J, et al. The Oncogenic Role of APC/C Activator Protein Cdc20 by an Integrated Pan-Cancer Analysis in Human Tumors. Front Oncol. 2021;11:721797.

56. Fernandez EV, Reece KM, Ley AM, et al. Dual targeting of the androgen receptor and hypoxia-inducible factor 1α pathways synergistically inhibits castration-resistant prostate cancer cells. Mol Pharmacol. 2015;87(6):1006-1012.

57. Shabsigh A, Ghafar MA, de la Taille A, et al. Biomarker analysis demonstrates a hypoxic environment in the castrated rat ventral prostate gland. J Cell Biochem. 2001;81(3):437-44.

58. Halin S, Hammarsten P, Wikström P, et al. Androgen-insensitive prostate cancer cells transiently respond to castration treatment when growing in an androgen-dependent prostate environment. Prostate. 2007;67(4):370-7.

59. Scher HI, Sawyers CL. Biology of progressive, castration-resistant prostate cancer: directed therapies targeting the androgen-receptor signaling axis. J Clin Oncol. 2005;23(32):8253-61.

60. Zhang Q, Huang R, Hu H, et al. Integrative Analysis of Hypoxia-Associated Signature in Pan-Cancer. iScience. 2020;23(9):101460.

61. Bhandari V, Li CH, Bristow RG, et al. Divergent mutational processes distinguish hypoxic and normoxic tumours. Nat Commun. 2020;11(1):737.

62. Ye Y, Hu Q, Chen H, et al. Characterization of Hypoxia-associated Molecular Features to Aid Hypoxia-Targeted Therapy. Nat Metab. 2019;1(4):431-444.

63. Nickols NG, Nazarian R, Zhao SG, et al. MEK-ERK signaling is a therapeutic target in metastatic castration resistant prostate cancer. Prostate Cancer Prostatic Dis. 2019 ;22(4):531-538.

64. Corno C, Arrighetti N, Ciusani E, et al. Synergistic Interaction of Histone Deacetylase 6- and MEK-Inhibitors in Castration-Resistant Prostate Cancer Cells. Front Cell Dev Biol. 2020; 8:610.

65. Lee M, Williams KA, Hu Y, et al. GNL3 and SKA3 are novel prostate cancer metastasis susceptibility genes. Clin Exp Metastasis. 2015;32(8):769-82.

Table 1. The relationships between SKA3 expression and clinicopathological features in prostate cancer patients in the TCGA database.

| Characteristic | Low expression of SKA3 (n=249) | High expression of SKA3 (n=250) | p |
| --- | --- | --- | --- |
| Age, n (%) |  |  | 0.080 |
| <=60 | 122 (54.5%) | 102 (45.5%) |  |
| >60 | 127 (46.2%) | 148 (53.8%) |  |
| T stage, n (%) |  |  | < 0.001 |
| T2 | 124 (65.6%) | 65 (34.4%) |  |
| T3 | 118 (40.4%) | 174 (59.6%) |  |
| T4 | 2 (18.2%) | 9 (81.8%) |  |
| N stage, n (%) |  |  | 0.036 |
| N0 | 171 (49.3%) | 176 (50.7%) |  |
| N1 | 28 (35.4%) | 51 (64.6%) |  |
| M stage, n (%) |  |  | 1.000 |
| M0 | 223 (49%) | 232 (51%) |  |
| M1 | 1 (33.3%) | 2 (66.7%) |  |
| Race, n (%) |  |  | 0.101 |
| Asian | 4 (33.3%) | 8 (66.7%) |  |
| Black or African American | 35 (61.4%) | 22 (38.6%) |  |
| White | 202 (48.7%) | 213 (51.3%) |  |
| Residual tumor, n (%) |  |  | 0.006 |
| R0 | 173 (54.9%) | 142 (45.1%) |  |
| R1 | 59 (39.9%) | 89 (60.1%) |  |
| R2 | 2 (40%) | 3 (60%) |  |
| Zone of origin, n (%) |  |  | 0.348 |
| Central Zone | 2 (50%) | 2 (50%) |  |
| Overlapping / Multiple Zones | 50 (39.7%) | 76 (60.3%) |  |
| Peripheral Zone | 69 (50.4%) | 68 (49.6%) |  |
| Transition Zone | 3 (37.5%) | 5 (62.5%) |  |
| PSA (ng/ml), n (%) |  |  | 0.102 |
| <4 | 214 (51.6%) | 201 (48.4%) |  |
| >=4 | 9 (33.3%) | 18 (66.7%) |  |
| Gleason score, n (%) |  |  | < 0.001 |
| 6 | 36 (78.3%) | 10 (21.7%) |  |
| 7 | 142 (57.5%) | 105 (42.5%) |  |
| 8 | 28 (43.8%) | 36 (56.2%) |  |
| 9 | 42 (30.4%) | 96 (69.6%) |  |
| 10 | 1 (25%) | 3 (75%) |  |
| PFI event, n (%) |  |  | < 0.001 |
| Alive | 218 (53.8%) | 187 (46.2%) |  |
| Dead | 31 (33%) | 63 (67%) |  |

SKA3: spindle and kinetochore-associated complex subunit 3; TCGA: the Cancer Genome Atlas; PFI: progress free interval.

**Figure Legends**

Figure 1. The correlation analysis between SKA3 expression and clinical parameters.


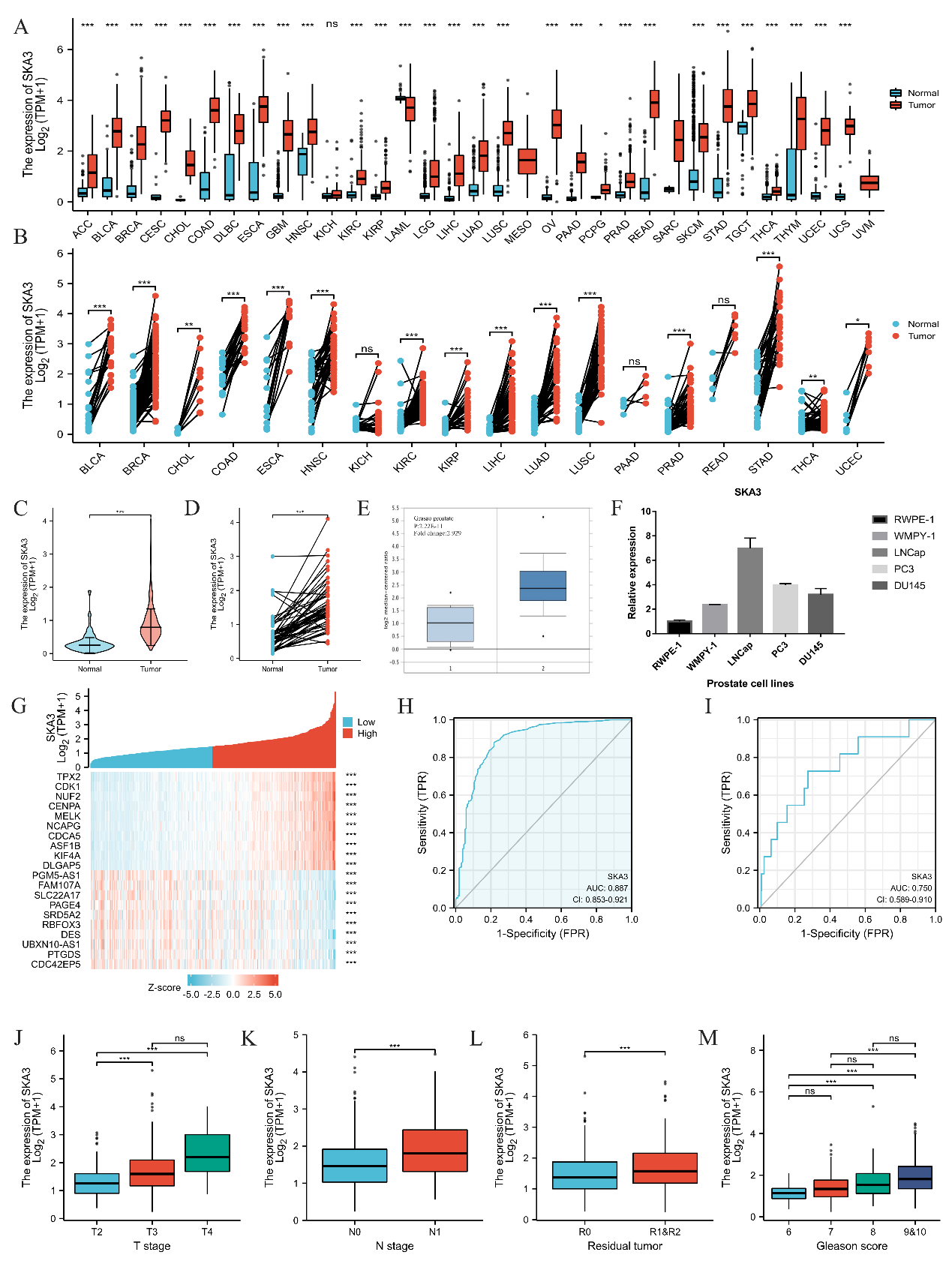
(A) the differential expressions of SKA3 in pan-cancer level using non-paired samples; (B) the differential expressions of SKA3 in pan-cancer level using paired samples; (C) the differential expressions of SKA3 in PCA using non-paired samples; (D) the differential expressions of SKA3 in PCA using paired samples; (E) the differential expressions of SKA3 in PCA in Oncomine database; (F) Relative expression of SKA3 among prostate normal and tumor cell lines; (G) heatmap showing top 20 related genes of SKA3; (H) the ROC curve of SKA3 distinguishing tumor from normal; (I) the ROC curve of SKA3 distinguishing T2-3 from T4; (J) comparison among T stages for SKA3 expression; (K) comparison among between N0 and N1 stage for SKA3 expression; (L) comparison among between R0 and R1-2 for SKA3 expression; (M) comparison among Gleason score for SKA3 expression. ROC= receiver operating characteristic curve. R=residual tumor.

Figure 2. The relationship between SKA3 expression and prognostic assessment in PCA patients, and SKA3-related functional analysis.


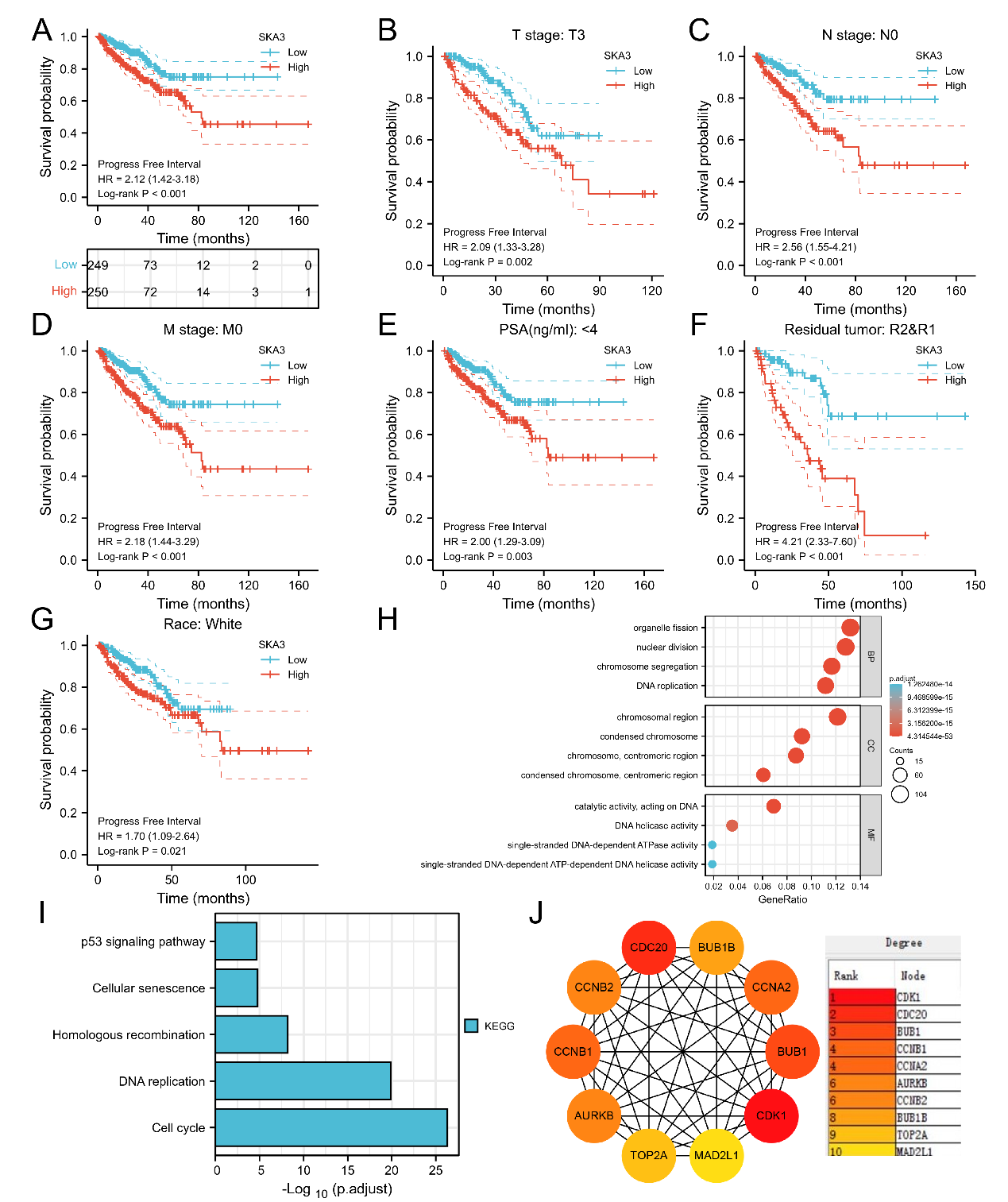
(A) Kaplan-Meier curve showing progress free survival of high and low expression of SKA3; (B) Kaplan-Meier curve showing progress free survival of high and low expression of SKA3 in patients with T3 stage; (C) Kaplan-Meier curve showing progress free survival of high and low expression of SKA3 in patients with N0 stage; (D) Kaplan-Meier curve showing progress free survival of high and low expression of SKA3 in patients with M0 stage; (E) Kaplan-Meier curve showing progress free survival of high and low expression of SKA3 in patients with PSA < 4 ng/ml; (F) Kaplan-Meier curve showing progress free survival of high and low expression of SKA3 in patients with positively residual tumor; (G) Kaplan-Meier curve showing progress free survival of high and low expression of SKA3 in white patients; (H) The GO analysis of SKA3 and its co-expressed genes; (I) The KEGG analysis of SKA3 and its co-expressed genes; (J) The top 10 hub genes of SKA3. GO= Gene ontology; KEGG= Kyoto Encyclopedia of Genes and Genome.

Figure 3. The differential expressions of the top 10 hub genes and their relationship with prognosis in PCA patients.


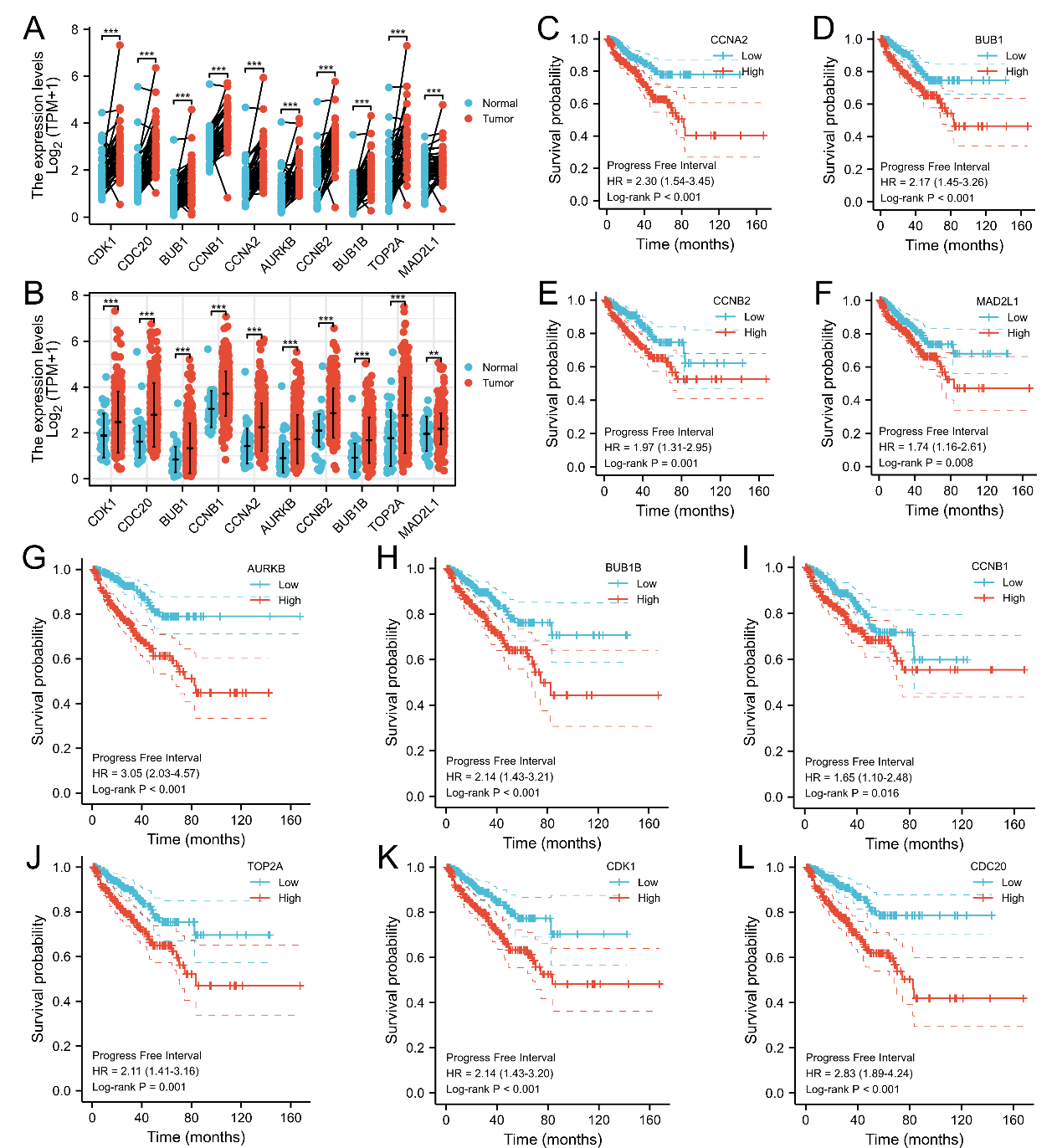
(A) the differential expressions of the top 10 hub genes in PCA using paired samples; (B) the differential expressions of the top 10 hub genes in PCA using non-paired samples; (C) Kaplan-Meier curve showing progress free survival of high and low expression of CCNA2; (D) Kaplan-Meier curve showing progress free survival of high and low expression of BUB1; (E) Kaplan-Meier curve showing progress free survival of high and low expression of CCNB2; (F) Kaplan-Meier curve showing progress free survival of high and low expression of MAD2L1; (G) Kaplan-Meier curve showing progress free survival of high and low expression of AURKB; (H) Kaplan-Meier curve showing progress free survival of high and low expression of BUB1B; (I) Kaplan-Meier curve showing progress free survival of high and low expression of CCNB1; (J) Kaplan-Meier curve showing progress free survival of high and low expression of TOP2A; (K) Kaplan-Meier curve showing progress free survival of high and low expression of CDK1; (L) Kaplan-Meier curve showing progress free survival of high and low expression of CDC20.

Figure 4. The results of gene set enrichment analysis, and cancer-related pathways of SKA3 and its co-expressed genes.


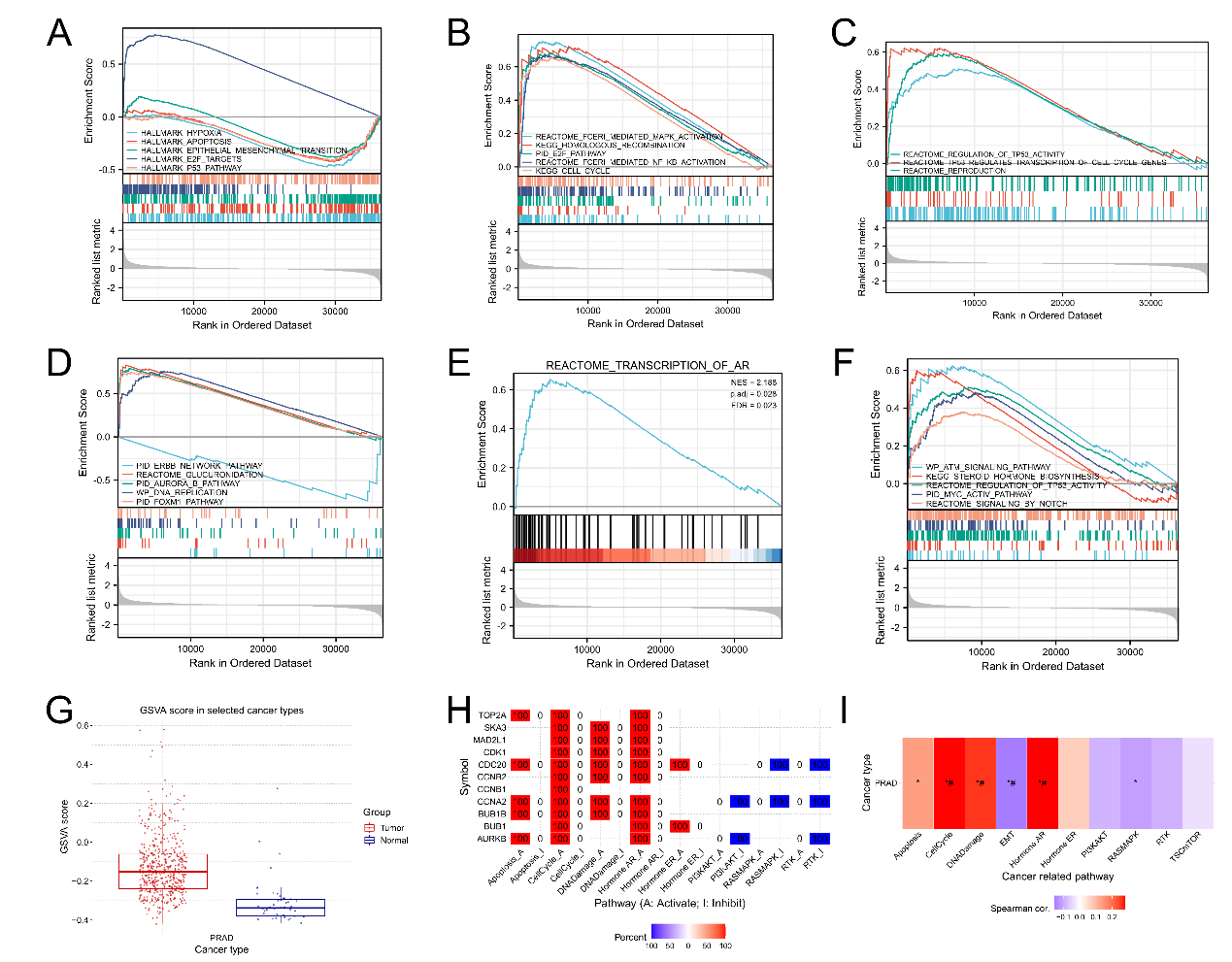
(A-F) the results of Gene Set Enrichment Analysis; (G) comparison between tumor and normal for GSVA score; (H) the pathways of SKA3 and its top 10 hub genes; (I) the pathways of the genset consisted with SKA3 and its hub genes. GSVA= Gene set variation analysis. *: p value ≤ 0.05; #: FDR ≤ 0.05.

Figure 5. The immune infiltration and drug sensitivity of SKA3 and its co-expressed genes.


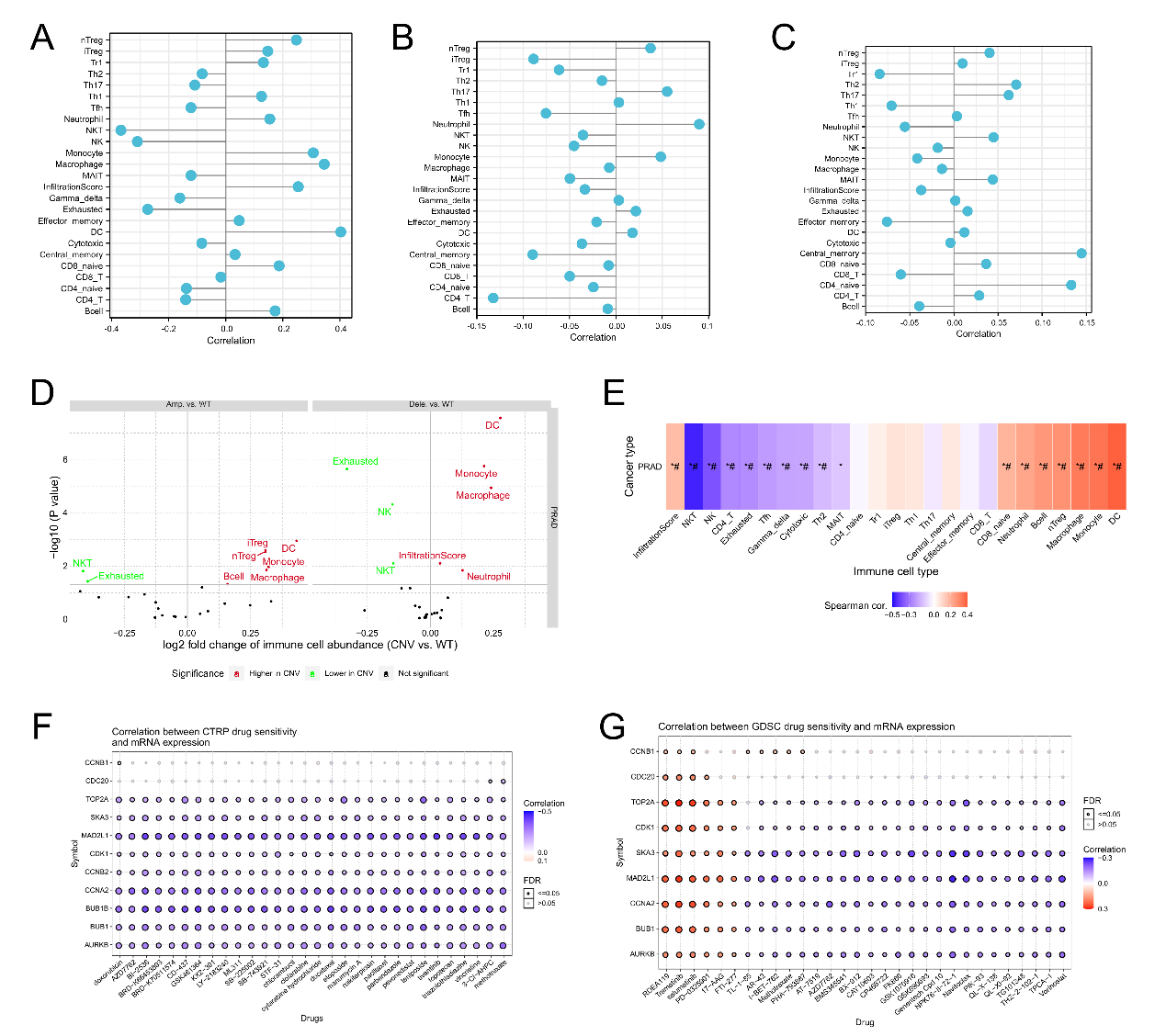
(A) correlation between SKA3 mRNA expression and immune infiltration; (B) correlation between SKA3 CNV and immune infiltration; (C) correlation between SKA3 methylation and immune infiltration; (D) the difference of immune infiltration between geneset CNV groups; (E) the correlation between immune cell infiltrates and GSVA enrichment score in the PCA; (F) the correlation between gene expression and the sensitivity of GDSC drugs (top 30) in pan-cancer; (G) the correlation between gene expression and the sensitivity of CTRP drugs (top 30) in pan-cancer. CNV= copy number variation; GDSC= genomics of drug sensitivity in cancer; CTRP= the cancer therapeutics response portal; PCA= prostate adenocarcinoma. *: p value ≤ 0.05; #: FDR ≤ 0.05.

Figure 6. The related predictive nomogram of progress free interval, and the relationship between SKA3 expression and hypoxia-related genes in PCA patients.


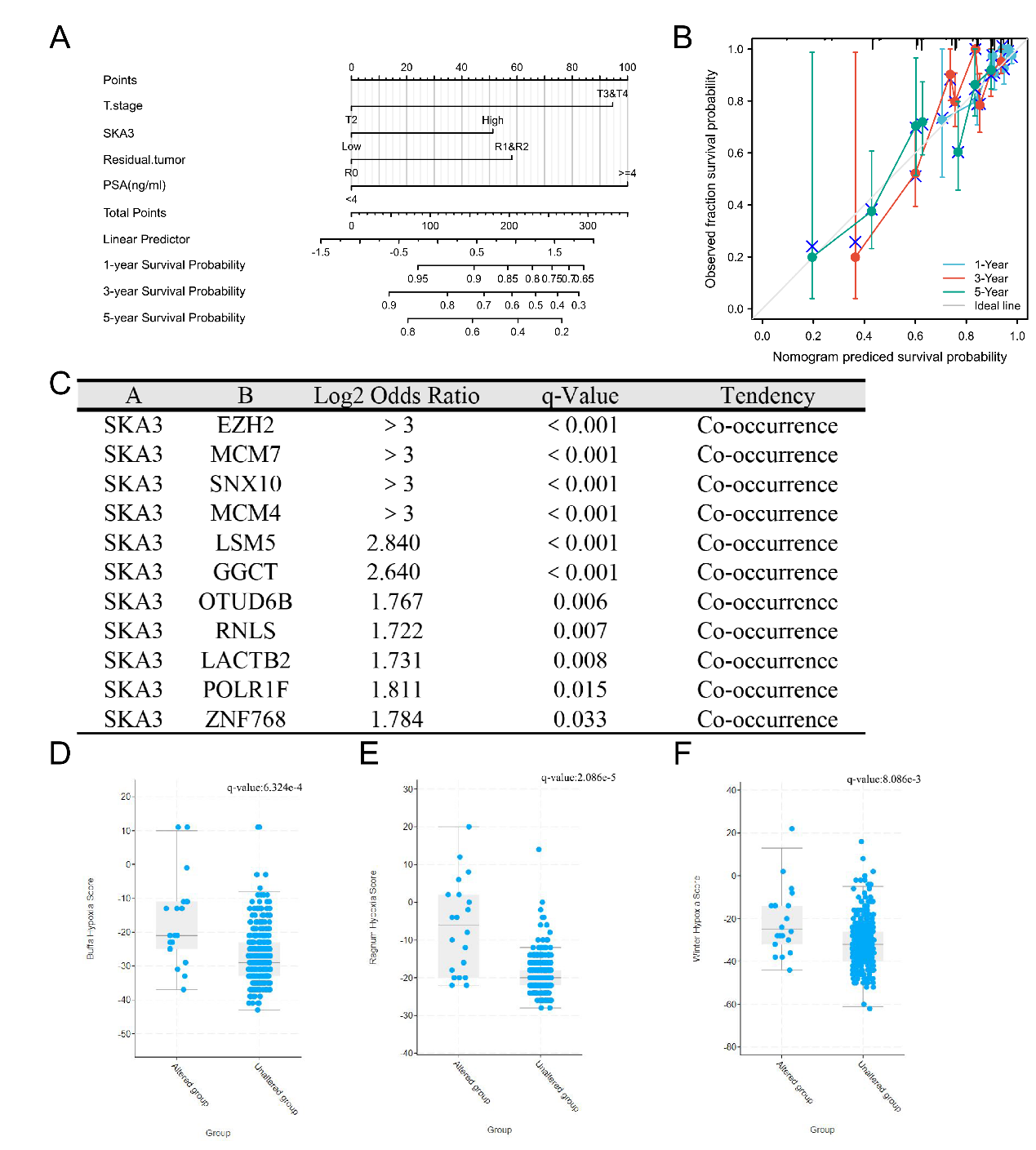
(A) the nomogram plot; (B) calibration plot; (C) analysis of mutual exclusivity for SKA3 and hypoxia-related genes; (D) comparison between SKA3 altered group and unaltered group for Buffa hypoxia score; (E) comparison between SKA3 altered group and unaltered group for Ragnum hypoxia score; (F) comparison between SKA3 altered group and unaltered group for Winter hypoxia score. PCA= prostate adenocarcinoma.

Figure 7. Cell proliferation.


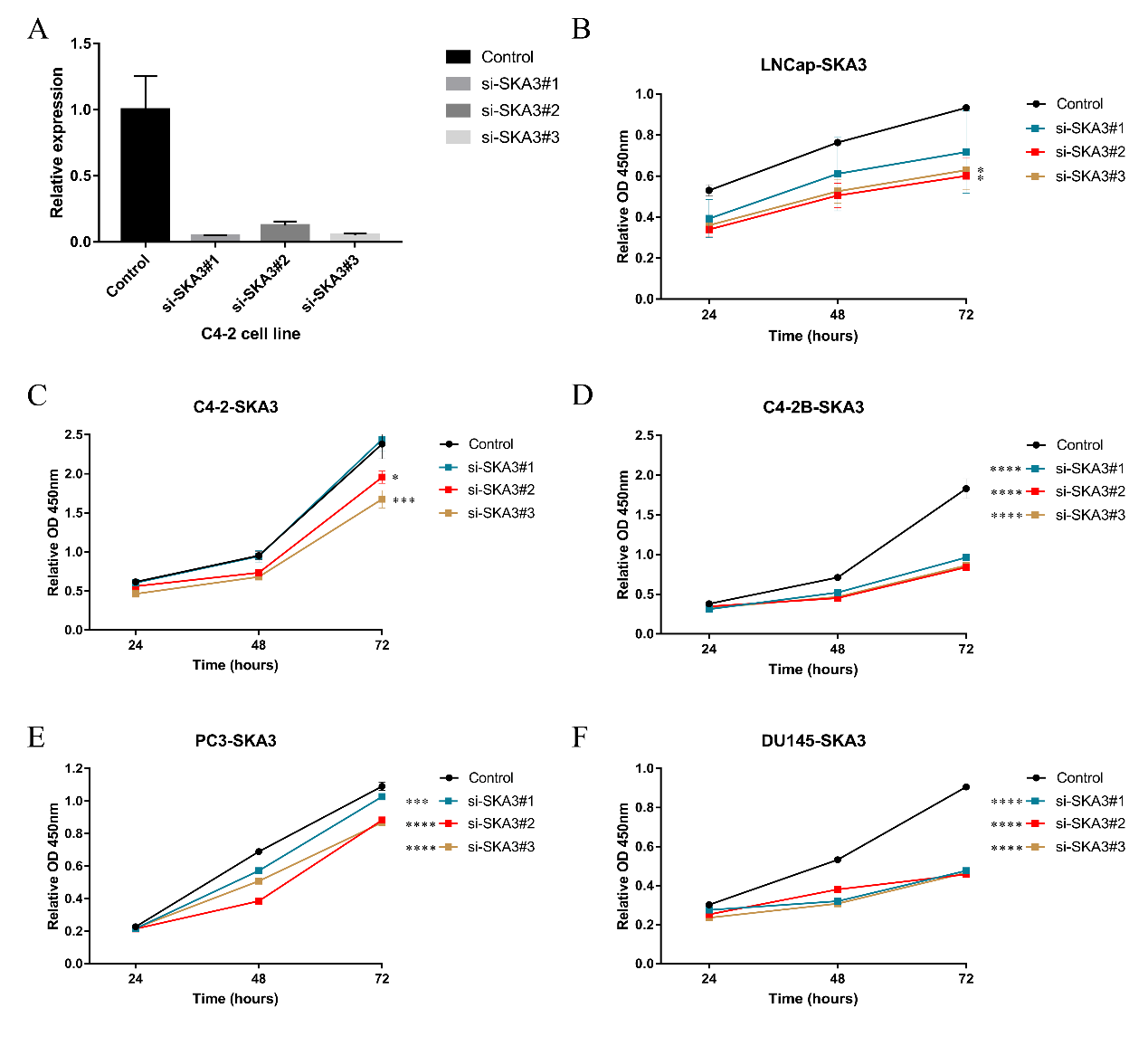


(A) RT-qPCR results of SKA3 siRNAs; (B) effect of SKA3 siRNAs on LNCap using CCK8 assay; (C) effect of SKA3 siRNAs on C4-2 using CCK8 assay; (D) effect of SKA3 siRNAs on C4-2B using CCK8 assay; (E) effect of SKA3 siRNAs on PC3 using CCK8 assay; (F) effect of SKA3 siRNAs on DU145 using CCK8 assay.

Supplementary figure 1. The genetic alteration of SKA3 and its co-expressed genes in PCA patients.


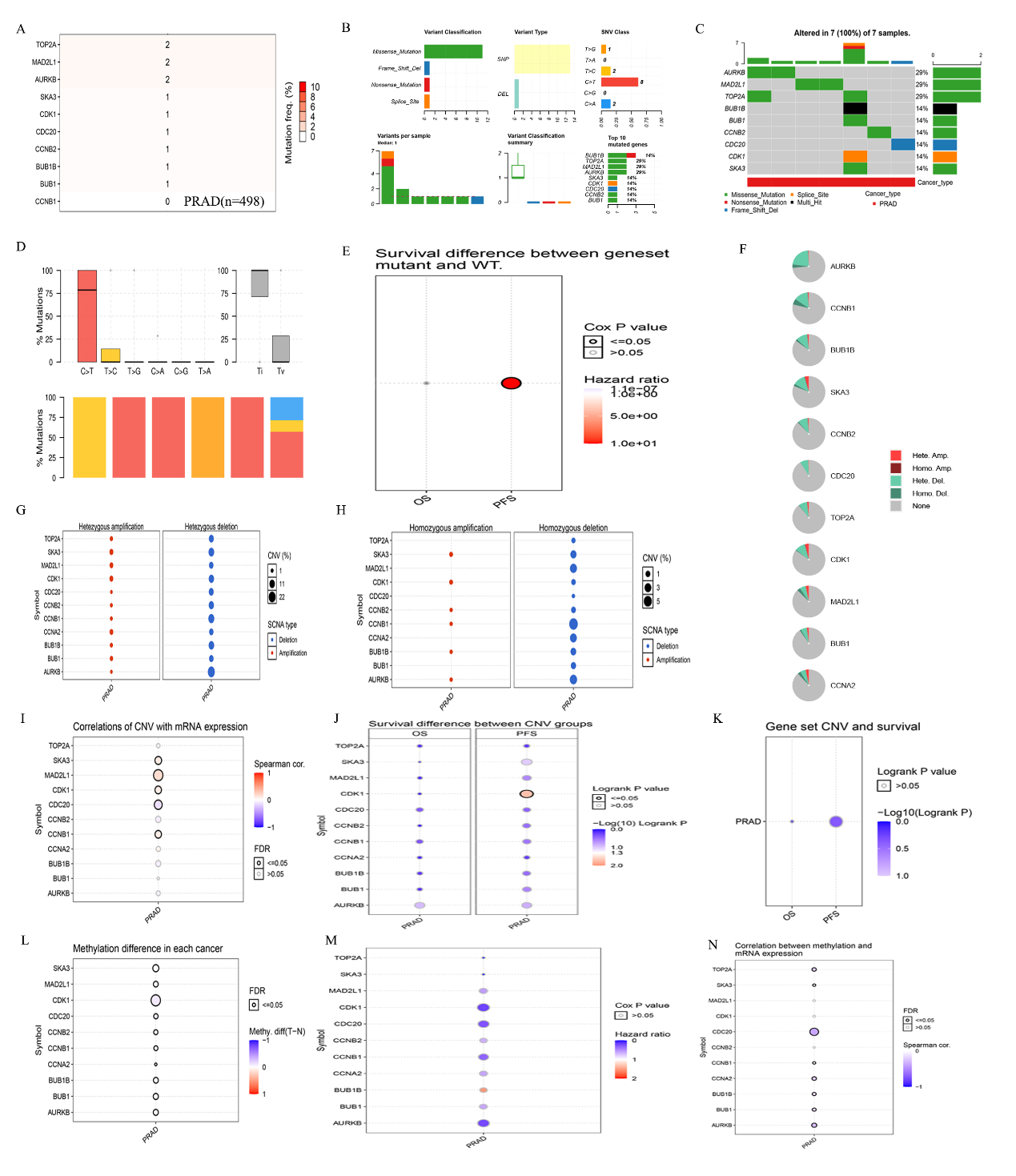


(A) the profile of SNV of the inputted gene set in the PCA; (B) the SNV classes of inputted gene set in the PCA; (C) Oncoplot providing the situation of the SNV of inputted gene set in the PCA; (D) the transitions(Ti) and transversions(Tv) classification of the SNV of inputted gene set in the PCA; (E) the survival difference between gene set mutant (effective) and wide type in the PCA; (F) Pie plot summarizing the CNV of inputted genes in the PCA; (G) the profile of heterozygous CNV of inputted genes in the PCA; (H) the profile of homozygous CNV of inputted genes in the PCA; (I) the correlations between CNV and mRNA expression in the PCA; (J) the difference of survival between CNV and wide type in the PCA; (K) the profile of survival between gene set CNV groups in the PCA; (L) the methylation difference between tumor and normal samples of inputted genes in the PCA; (M) the OS difference between higher and lower methylation groups in the PCA; (N) the profile of correlations between methylation and mRNA expression of inputted genes in the PCA. OS=overall survival; PFS=progression free survival; SNV= single nucleotide variation; CNV= copy number variation; PCA= prostate adenocarcinoma.

Note: Supplementary figure 1B-D: the analysis is being performed on the 7 samples containing mutations in the PCA dataset.
